# Hemoglobin level, a prognostic factor for nasal extranodal natural killer/T-cell lymphoma patients from stage I to IV: A validated prognostic nomogram

**DOI:** 10.1038/s41598-017-11137-9

**Published:** 2017-09-08

**Authors:** Jianzhong Cao, Shengmin Lan, Liuhai Shen, Hongwei Si, Huan Xiao, Qiang Yuan, Xue Li, Hongwei Li, Ruyuan Guo

**Affiliations:** 1 Department of Radiotherapy, Shanxi Cancer Hospital and Institute, Affiliated Hospital of Shanxi Medical University, Shanxi, 030013 China; 2 Department of Nuclear Medicine, the First Affiliated Hospital of Anhui Medical University, Hefei, Anhui Province 230022 China

## Abstract

Although nasal extranodal natural killer/T-cell lymphoma (nasal ENKL) shares some prognostic factors with other lymphomas, seldom studies had explored the prognostic value of hemoglobin. The ENKL cases in stage I–IV during 2000 to 2015 were collected from two medical centers (group A, n = 192), and were randomly divided into the group B (n = 155) and C (n = 37). Although the significant factors identified by the univariate analysis differed between the group A and B, the multivariate Cox regression indicated the same factors. C-index of the model was slightly better than Yang’s, but its integrated Brier score (IBS) was obviously lower than Yang’s both in the group A and B. Additionally, minimal depth of random survival forest (RSF) classifier confirmed that the prognostic ability of hemoglobin was better than age both in the group A and B. In the calibration of the nomogram, the predicted 3-year or 5-year OS of our nomogram well agreed with the corresponding actual OS. In conclusion, Hemoglobin is a prognostic factor for nasal ENKL patients in stage I - IV, and integrating it into a validated prognostic nomogram, whose generalization error is the smallest among the evaluated models, can be used to predict the patients’ outcome.

## Introduction

Nasal extranodal natural killer/T-cell lymphoma (ENKL) is a rare subset of lymphomas, and is relatively prevalent in Asia^1^. The patients with the aggressive disease often have good performance status at the first visit^2^, but frequently exhibit multidrug resistance (MDR) and do not well respond to the anthracycline-based regimens (CHOP)^3^.

Currently, discriminating the patients with unfavorable prognostic factors is important for the selection of treatment modalities^4^. According to the study from Yang *et al*.^4^, for stage IE patients without risk-factors (age < 60 y, Eastern Cooperative Oncology Group performance status <2, normal LDH, Ann Arbor stage I, and no primary tumor invasion), radiotherapy (RT) alone could achieve a 5-year overall survival rate (OS rate) of 88.8%. For stage IE patients with one of these risk-factors, the treatment of combined RT and chemotherapy (CT) could achieve a 5-year OS rate of 72.2%.

Low hemoglobin level is associated with poor outcome of some lymphomas, such as Hodgkin and diffuse large B-Cell lymphoma^5, 6^. Although nasal ENKL is classified as a type of lymphoma^7^, and shares some prognostic factors with others, such as International Prognostic Index (IPI) and lactic dehydrogenase level (LDH)^8, 9^, seldom studies had evaluated the prognostic ability of hemoglobin for nasal ENKL. Therefore, we designed this study to explore its prognostic ability for the patients in stage I–IV, and to integrate it into a prognostic nomogram.

## Methods

### Patients, Treatment and Follows

At the Shanxi Cancer Hospital and Institute (SCHI) and the First Affiliated Hospital of Anhui Medical University (FAHAMU), nasal ENKL cases between 2000 and 2015 were collected according to the morphological and immunohistological criteria of the World Health Organization classification^10^. Before any treatment, patient information included history taking, physical and laboratory examinations, and results of computerized tomography. Hemoglobin levels were classified into lower or higher than 120 g/L. To minimize migrations of staging techniques through a time-span of 15 years, only computerized tomography series of PET/CT scans were used to stage and evaluate the diseases (n = 17). Our protocol was approved by the ethics committee at SCHI and FAHAMU.

Because tumor heterogeneity affects the robustness of predictors^11^, some re-sampling approaches are used to ensure our model can be applied in other cohorts of patients. Using SPSS software (version 10.01), 20% of all recruited patients (group A) were randomized into the group C, and the rest was as the group B. Both the group A and B were used to develop prognostic model for guaranteeing its repeatability. And then, the group B and C were used to validate the model for assuring its reliability. The approach was similar to the external validation procedure, i.e. the developed model (from group B) was validated in another cohort of patients (group C).

Additionally, using the bootstrap method, a 10 fold cross-validation was used to test the generalization ability of the model^12^. The patients (group A and B) were equally and randomly divided into10 subsets. And then, the model was repeatedly trained and validated 10 times. Each time, the pooled data of 9 subsets were used to train the model, which was validated in the retained subset subsequently. The average error across 10 rounds (integrated Brier score, IBS) could estimate the error in generalizing the model in an independent dataset, and a lower IBS indicated a better model^13^.

According to the paradigm of the two hospitals, CT was the first-line treatment, and only the poor responders would receive RT. After the publication of the study by Li *et al*.^14^ in 2006, more patients received RT than before; however, RT was still not the first-line treatment. From medical records or by telephone, all patients were followed to the end of August 2016. Overall survival (OS) was measured from the day of diagnosis to death from any cause.

### Prognostic Model and Validation

Separately using the data of the group A and B, the significance of prognostic factors against OS was univariately identified by the Kaplan–Meier and the Log-rank test (P < 0.05). And then, the multivariate Cox regression was used to select the qualified factors against OS from the significant ones. At last, the same factors between the group A and B were used to develop our model.

**Table 1:** Patient characteristics.

Characteristic		Group A	Group B	Group C	P
		n = 192 (%)	n = 155 (%)	N = 37 (%)	
Gender	Male	155 (80.7)	122 (78.7)	33 (89.2)	0.146
	female	37 (19.3)	33 (21.3)	4 (10.8)	
Age (y)	≤60	169 (88.0)	137 (88.4)	32 (86.5)	0.749
	>60	23 (12.0)	18 (11.6)	5 (13.5)	
B symptoms	Absent	119 (62.0)	99 (63.9)	20 (54.1)	0.269
	Present	73 (38.0)	56 (36.1)	17 (45.9)	
Waldeyer’s ring	Absent	27 (14.1)	131 (84.5)	31 (83.8)	0.912
	Present	165 (85.9)	24 (15.5)	6 (16.2)	
PTI	Absent	110 (57.3)	88 (56.8)	22 (59.5)	0.767
	Present	82 (42.7)	67 (43.2)	15 (40.5)	
LDH	Normal	140 (72.9)	112 (72.3)	28 (75.7)	0.674
	Abnormal	52 (27.1)	43 (27.7)	9 (24.3)	
Hemoglobin	≥120 g/L	161 (83.9)	131 (84.5)	30 (81.1)	0.610
	<120 g/L	31 (16.1)	24 (15.5)	7 (18.9)	
Distant metastasis	Absent	175 (91.1)	140 (90.3)	35 (94.6)	0.411
	Present	17 (8.9)	15 (9.7)	2 (5.4)	
Ann Arbor stage	I	117 (60.9)	95 (61.3)	22 (59.5)	0.950
	II	48 (25.0)	38 (24.5)	10 (27.0)	
	III-IV	27 (14.1)	22 (14.2)	5 (13.5)	
ECOG PS	0–1	142 (74.0)	112 (72.3)	30 (81.1)	0.272
	2–4	50 (26.0)	43 (27.7)	7 (18.9)	
Treatment	CT alone	76 (39.6)	64 (41.3)	12 (32.4)	0.143
	RT alone	9 (4.7)	9 (5.8)	0 (0.0)	
	combined	107 (55.7)	82 (52.9)	25 (67.6)	

P: The significant between group B and C. PTI: Primary tumor invasion. ECOG PS: Eastern Cooperative Oncology Group performance status.

To validate IPI, Korean prognostic index (KPI), Yang’s^9^, and our models, their discriminatory ability of the three groups was compared by C-index (mean ± SE), which was similar to the area under the receiver operating curve (ROC) of the models. After that, the nomogram of our model was built, and calibration plots of the model were constructed between the predicted and the observed survival probabilities. A better model would have a C-index closing to 1, and a calibration curve closing to the line passing through the original point with a slope of 1.

Additionally, to evaluate the prognostic ability of factors involved in these models, especially age and hemoglobin, an indicator of random survival forest (RSF) classifier was used. Although RSF is not so popularly used as the Cox multivariate regression, it is a more accurate method for analyzing survival data. Minimal depth is an indicator of RSF classifier to evaluate the prognostic ability of each factor. A smaller minimal depth of a factor is, the more ability it has on prognosis^15, 16^.

Data were analyzed with the SPSS statistical software (version 10.01) and the R Project software package (version 3.3.1). A two sides of P < 0.05 was considered as the significant level.

### Ethics approval and informed consent

Our protocol was approved by the ethics committee of the Shanxi Cancer Hospital and Institute, and the First Affiliated Hospital of Anhui Medical University. The study was conducted in accordance with the relevant guidelines and regulations. Informed consent was obtained from all participants according to the institutional guidelines.

### Data availability statement

The datasets used and/or analyzed during the current study are available from the corresponding author on reasonable request.

## Results

### Patient Characteristics and Treatments

The median ages of the group A (n = 192), B (n = 155) and C (n = 37) were 42.8 y (9–79 y), 42.7 y (9–79 y) and 43.0 y (17–74 y), respectively. Patient characteristics are listed in Table 1, and are well balanced between the group B and C.

**Figure 1:**
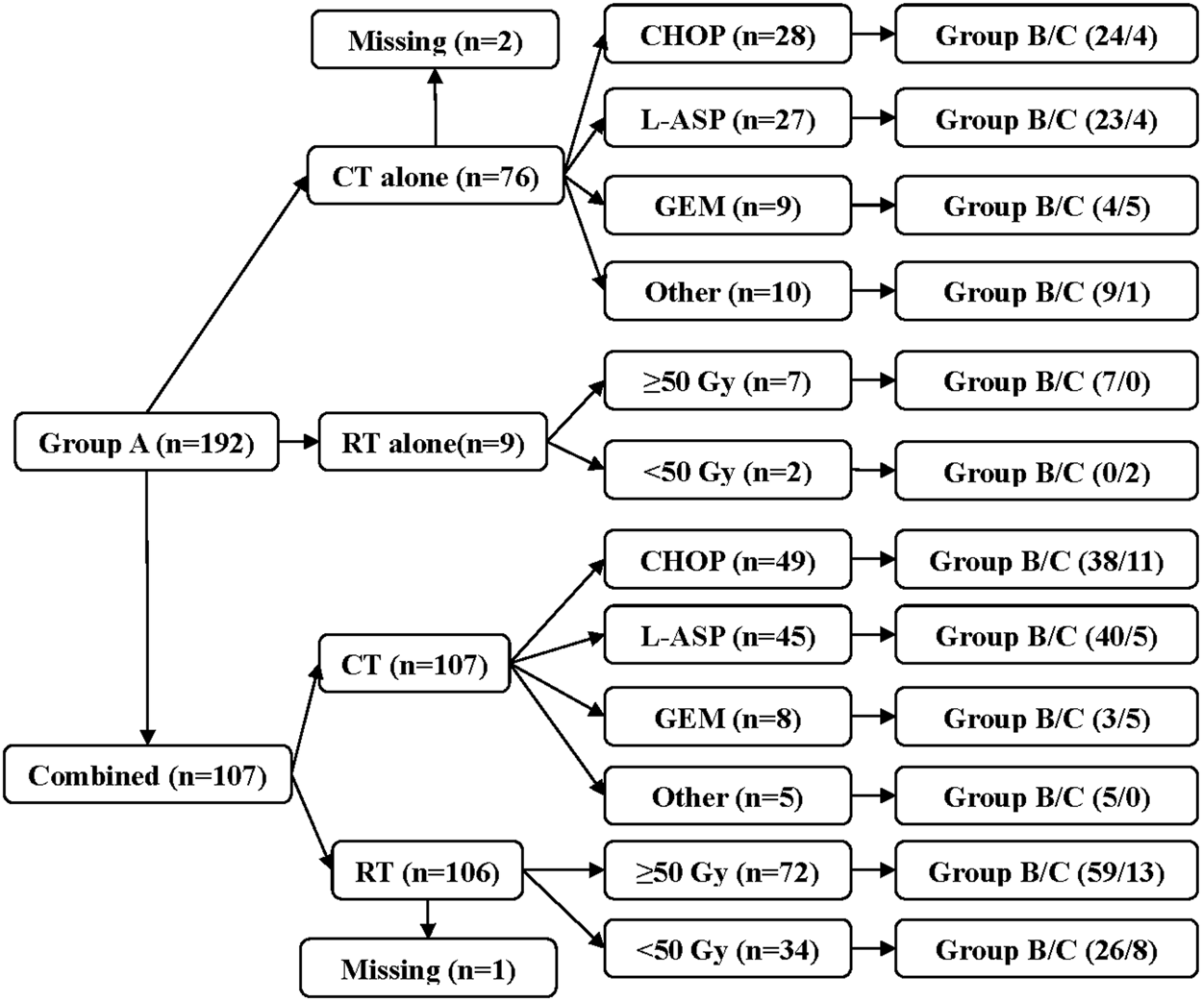
Patient treatments.

Patient treatments are presented in Fig. 1. Among the patients received CT alone (n = 76), the CHOP or CHOP like, L-ASP, GEM, and other regimens were administered 4–7 cycles (n = 28, median 6), 4–8 cycles (n = 27, median 6), 4–8 cycles (n = 9, median 6) and 4–7 cycles (n = 10, median 6), respectively. Among the patients received combined treatment (n = 107), CHOP or CHOP like, L-ASP, GEM and other regimens were administered 4–8 cycles (n = 49, median 6), 4–9 cycles (n = 45, median 6), 4–8 cycles (n = 8, median 6) and 4–7 cycles (n = 5, median 6), respectively. Between the group B and C, neither regimens (CHOP vs non-CHOP, *x* ^2^ = 0.002, P = 0.966) nor CT cycles (*x* ^2^ = 11.757, P = 0.302) existed significant difference.

RT (n = 115) was given by 6-MV linear accelerators (Varian), and the median dose was 50 Gy (range 8–70 Gy). The most patients (n = 75) received conventional radiotherapy (46/75 patients ≥ 50 Gy), and others (n = 40) received intensity modulated radiotherapy or three dimensional conformal radiation therapy (33/40 patients ≥ 50 Gy). Between the group B and C, radiation dose of 66/92 and 13/23 patients were higher than 50 Gy (*x* ^2^ = 1.981, P = 0.159), and 60/75 and 32/40 patients received conventional RT (*x* ^2^ = 0.000, P = 1.000), respectively.

### Outcome and Prognostic Model

By the end of August 2016, the median observation times on the group A, B and C were 24.0 months (range 0–142), 23.0 months (range 0–131) and 27.0 months (range 1–142), respectively. Eighty-one patients died within 0.0 to 127.0 months (median 12.0 months). The results of univariate analysis of the group A and B are presented in Table 2, and only the significance of the factor of distant metastasis is different between the two groups. Additionally, the factor of treatment was significant for OS in both groups.

**Table 2:** Results of univariate analysis.

		Group A (n = 192)			Group B (n = 155)		
		5-year OS rate (%)	Median (months)	P value	5-year OS rate (%)	Median (months)	P value
B symptoms	Absent	46.1	53.9	0.751	48.5	54.4	0.936
	Present	51.0	66.1		48.8	45.6	
Gender	Male	48.2	54.2	0.636	0.519	60.4	0.090
	female	46.7	45.6		38.8	31.8	
Waldeyer’s	Absent	47.5	54.2	0.561	49.3	54.4	0.245
ring	Present	48.7	52.6		44.4	52.6	
Age (y)	≤60	47.8	54.2	0.205	47.8	54.2	0.408
	>60	47.4	24.8		51.6	82.4	
Distant	Absent	50.0	60.4	0.054	51.6	66.1	0.032
metastasis	Present	22.6	30.7		21.5	30.7	
Ann Arbor	I	57.1	129.4	0.000	59.4	NA	0.000
stage	II	26.2	25.8		26.4	25.8	
	III-IV	44.9	31.8		39.4	31.8	
PTI	Absent	61.3	NR	0.000	59.6	NR	0.001
	Present	28.7	25.3		33.3	27.7	
LDH	Normal	55.4	82.4	0.001	56.6	77.0	0.005
	Abnormal	22.6	33.2		24.4	33.4	
Hemoglobin	≥120 g/L	51.1	60.4	0.001	52.2	77.0	0.002
	<120 g/L	28.8	23.6		18.9	23.6	
ECOG PS	0–1	58.5	129.4	0.000	60.3	NR	0.000
	2–4	16.1	13.0		14.4	13.0	
Treatment	CT alone	38.1	35.7	0.042	40.1	33.7	0.031
	RT alone	50.0	23.5		50.0	23.5	
	combined	55.3	82.4		56.4	82.4	

PTI: Primary tumor invasion. ECOG PS: Eastern Cooperative Oncology Group performance status.

In the multivariate Cox regression of group A, treatment (Score = 1.644, P = 0.200) was excluded from the model, while Ann Arbor stage (OR = 1.389, P = 0.025), primary tumor invasion (OR = 2.004, P = 0.003), LDH (OR = 2.073, P = 0.003), hemoglobin (OR = 2.341, P = 0.003), and ECOG performance status (OR = 4.116, P = 0.000) were significant. For the group B, both distant metastasis (Score = 0.511, P = 0.475) and treatment (Score = 1.802, P = 0.180) were excluded. Therefore, the significant factors were same between the group A and B, and were Ann Arbor stage, primary tumor invasion (PTI), LDH, hemoglobin and Eastern Cooperative Oncology Group performance status (ECOG PS).

### Prognostic Model and Nomogram Validation

**Figure 2:**
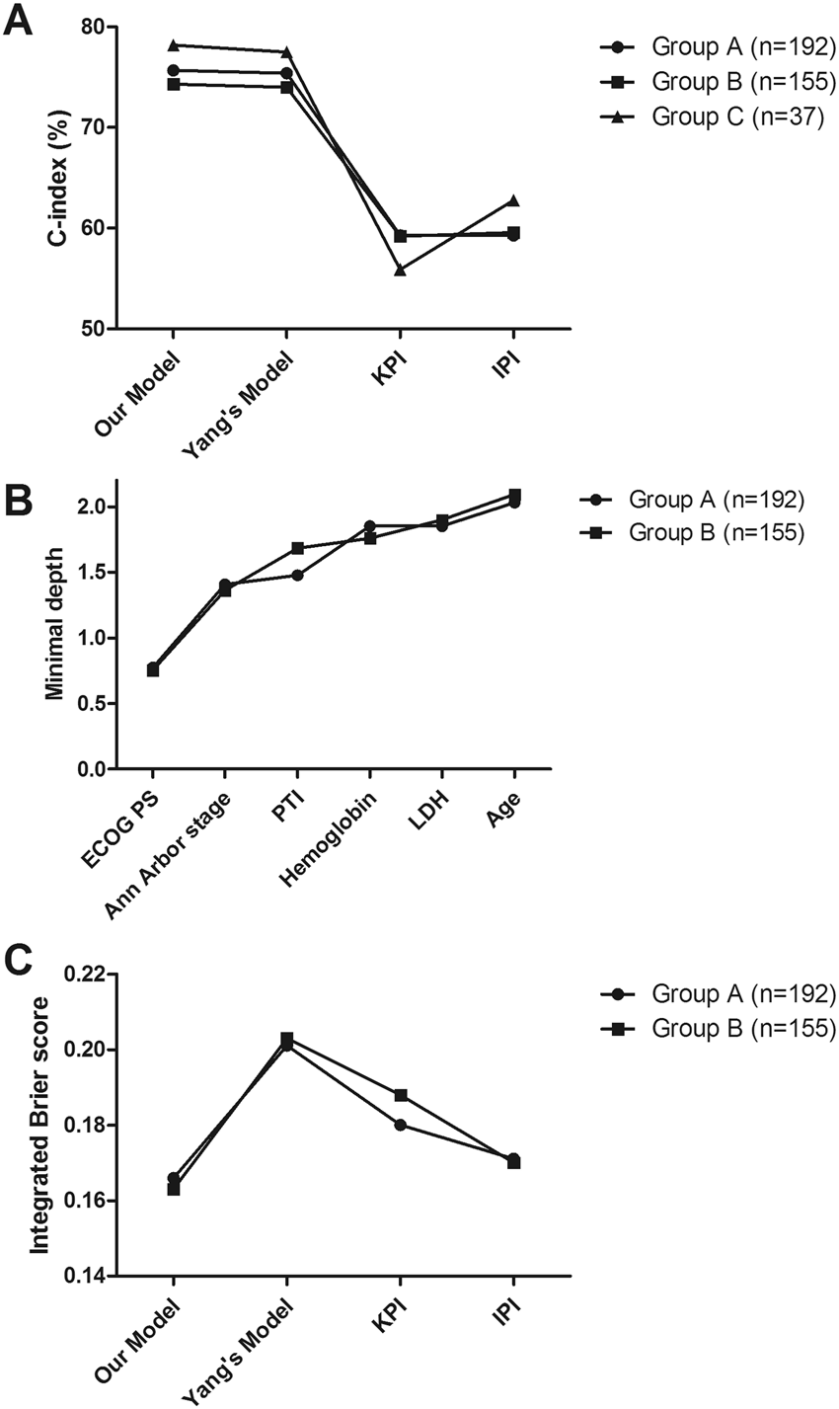
Evaluation of prognostic models and factors. (**A**) C-index of the models. (**B**) Minimal depth of factors involved in Yang’s model and ours. (**C**) Integrated Brier score of the models.

To validate prognostic models, C-index of IPI, KPI, Yang’s, and our model was separately calculated for the three groups (Fig. 2A), and indicated that ours and the Yang’s model were obviously better than IPI or KPI index. Furthermore, C-index of our model (mean ± SE) was slightly better than that of Yang’s in the group A (75.7% ± 4.4% vs. 75.4% ± 4.4%), B (74.3% ± 4.8% vs. 74.0% ± 4.8%) and C (78.2% ± 10.7% vs. 77.5% ± 10.6%).

**Figure 3:**
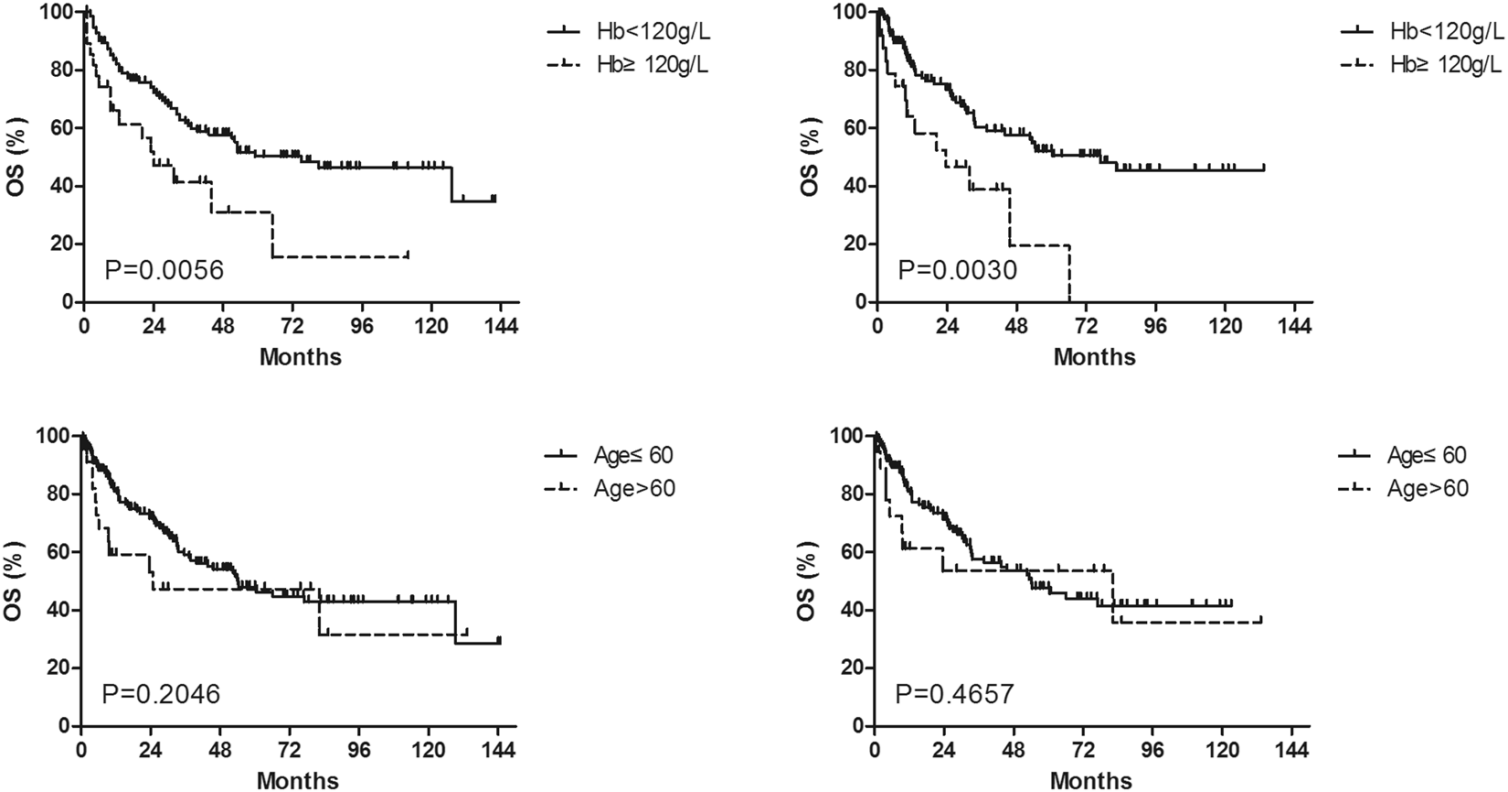
Survival plots of hemoglobin and age for OS in the group A and B. The left and right columns are those for the group A and B, respectively. The upper and lower rows are those for hemoglobin and age, respectively.

Because our model differed from Yang’s in the substitution of age with hemoglobin, survival plots of age and hemoglobin were compared (Fig. 3), and indicated that hemoglobin was better than age in both group A and B. The result was also confirmed by RSF classifier. Both in the group A and B, the minimal depth of hemoglobin were lower than that of age, and were even lower than that of LDH (Fig. 2B).

In the 10 fold cross-validation of the group A and B (Fig. 2C), our model’s IBS was obviously lower than others’, and the Yang’s was even the highest. Therefore, these results indicated that the generalization error of our model was the smallest among the evaluated models.

**Figure 4:**
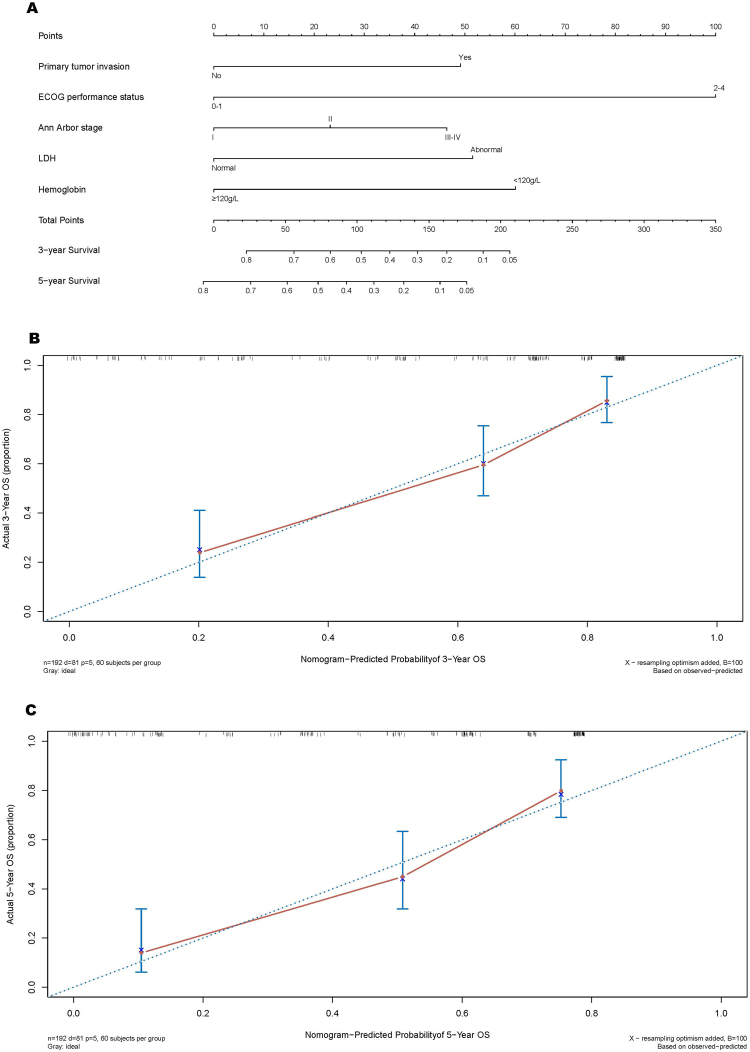
Nomogram and calibration of our model. (**A**) Nomogram. (**B,C**) are the calibration plots for 3-year and 5-year prediction, respectively.

In the group A, the nomogram of our model for predicting 3-year and 5-year OS is plotted in Fig. 4A. To calibrate the nomogram, the predicted 3-year and 5 year OS was separately plotted against corresponding actual OS (Fig. 4B and C). On the plots, the curve between predicted and actual OS closes to the line passing the original point with a slope of 1, which indicates the good agreement between them.

## Discussion

Our study indicates that hemoglobin level is a prognostic factor for the patients with nasal extranodal natural killer/T-cell lymphoma in stage I - IV, and the validated prognostic nomogram (Ann Arbor stage, PTI, LDH, hemoglobin, and ECOG PS) can be used to predict the outcome of the patients. Although, among the evaluated models, ours just slightly improves C-index, its generalization error is the smallest.

Classified as a kind of lymphoma, nasal ENKL shared some prognostic factors with other lymphomas, such as IPI and LDH level^8, 9^. Previously, using multivariate Cox regression, several studies tried to relate hemoglobin to the prognosis of the patients. However, because of the limited cases, Ma^17^ and Kim^18^ (n = 64 and 62, respectively) failed. In the study from Xu *et al*.^19^ (n = 170), according to hemoglobin levels, the recruited patients were grouped (threshold value 100 g/L, vs. 120 g/L of our study). In the univariate analysis, the factor of hemoglobin was significant against progression free survival (P = 0.034), but not significant against OS (P = 0.057). In a cohort of 321 patients, Wang *et al*.^20^ found that hemoglobin, ECOG PS, age, LDH and Ann Arbor stage were significant factors for both progression free survival and OS, but they did not validate the results in another group of patients^20^. Therefore, we confirmed their results that hemoglobin level was a prognostic factor for nasal ENKL patients.

Compared to multivariate Cox regression, random survival forest classifier is better in modeling non-linear effects and complex interactions among factors. Furthermore, the classifier can provide indicators, such as minimal depth, to evaluate the prognostic ability of each factor^21^. As indicated by the depth of RSF classifier, hemoglobin was better than age in predicting the outcome of the patients. Additionally, the results were also confirmed by the survival plots of age and hemoglobin (Fig. 3).

Therefore, the factor of age in Yang’s model, which regressed from the patients in stage I and II^9^, was substituted by hemoglobin, and the substitution could slightly improve the C-index of the model. Furthermore, as indicated by the IBS of 10 fold cross-validation, the generalization error of our model was the smallest among the evaluated models, especially was obviously better than Yang’s. It should be noted that, for the comparison of models, we re-regressed Yang’s model, which differed from its origin in the scoring points of factors. Therefore, the re-regression made up the difference of C-index between Yang’s and our model.

For diffuse large B-cell lymphoma and non-Hodgkin lymphoma, hemoglobin < 120 g/L is a frequent sign at diagnosis, and interleukin-6 plays a vital role in its development^8^. Because it had been reported that both interleukin-9 and −10 related to the poor prognosis of the nasal ENKL patients, the underlining mechanism might be that interleukins act as growth factors of tumor cells and participate in the production of erythropoietin (EPO)^22, 23^. However, further studies should be conducted to confirm this hypothesis.

Besides the prognostic factors, there were other differences between Yang’s and our nomogram. In the Yang’s model^9^, Ann Arbor stage had the highest score, that was followed by ECOG PS, PTI, age and LDH. In contrast, the sequence of our nomogram was ECOG PS, hemoglobin, LDH, PTI and Ann Arbor stage. Additionally, Yang’s model was developed for stage I-II patients, and ours was for those from stage I to IV.

Besides the prognostic factors identified by previous studies, other powerful factors might be long non-coding RNAs (lnRNAs), microRNAs (miRNAs), and so on. The potential relation between these markers and nasal ENKL could be predicted by some computational models^24–26^. After verifying the markers in laboratory research, our future work would focus on their prognostic ability.

As a study with limited cases, we tried another way to validate our model. Firstly, the enrolled patients (n = 192, group A) were randomly divided into group B (n = 155) and C (n = 37). Subsequently, to validate the repeatability of our model, both the group A and B were separately used to develop prognostic models. And then, C-index of all groups was used to evaluate the discriminatory of models. The approach was similar to the external validation procedure, i.e. the developed model (from group B) was validated in another cohort of patients (group C). At least in this study, the prognostic ability of hemoglobin was coincided with the results from Wang *et al*.^20^, and indicated that the validation method might be useful for other studies with limited cases.

## Conclusions

Hemoglobin is a prognostic factor for nasal extranodal natural killer/T-cell lymphoma patients from stage I to IV, and integrating it into a validated prognostic nomogram, whose generalization error is the smallest among the evaluated models, can be used to predict the outcome of the patients.
